# Comprehensive intravascular imaging of atherosclerotic plaque *in vivo* using optical coherence tomography and fluorescence lifetime imaging

**DOI:** 10.1038/s41598-018-32951-9

**Published:** 2018-09-28

**Authors:** Min Woo Lee, Joon Woo Song, Woo Jae Kang, Hyeong Soo Nam, Tae Shik Kim, Sunwon Kim, Wang-Yuhl Oh, Jin Won Kim, Hongki Yoo

**Affiliations:** 1 Department of Biomedical Engineering, Hanyang University, Seoul, 04763 Republic of Korea; 2 Multimodal Imaging and Theranostic Lab, Cardiovascular Center, Korea University Guro Hospital, Seoul, 08308 Republic of Korea; 3 Department of Mechanical Engineering, KAIST, Daejeon, 34141 Republic of Korea; 4 Department of Cardiology, Korea University Ansan Hospital, Ansan, 15355 Republic of Korea

## Abstract

Comprehensive imaging of both the structural and biochemical characteristics of atherosclerotic plaque is essential for the diagnosis and study of coronary artery disease because both a plaque’s morphology and its biochemical composition affect the level of risk it poses. Optical coherence tomography (OCT) and fluorescence lifetime imaging (FLIm) are promising optical imaging methods for characterizing coronary artery plaques morphologically and biochemically, respectively. In this study, we present a hybrid intravascular imaging device, including a custom-built OCT/FLIm system, a hybrid optical rotary joint, and an imaging catheter, to visualize the structure and biochemical composition of the plaque in an atherosclerotic rabbit artery *in vivo*. Especially, the autofluorescence lifetime of the endogenous tissue molecules can be used to characterize the biochemical composition; thus no exogenous contrast agent is required. Also, the physical properties of the imaging catheter and the imaging procedures are similar to those already used clinically, facilitating rapid translation into clinical use. This new intravascular imaging catheter can open up new opportunities for clinicians and researchers to investigate and diagnose coronary artery disease by simultaneously providing tissue microstructure and biochemical composition data *in vivo* without the use of exogenous contrast agent.

## Introduction

Coronary artery disease (CAD) is the biggest cause of death worldwide^1^. Atherosclerosis, which is characterized by the formation of atherosclerotic plaque on the coronary artery wall, is the most common form of CAD^2^. Because most acute cardiac events, such as fatal myocardial infarction and sudden cardiac death, are caused by thrombotic complications following plaque rupture, it is essential to assess the vulnerability of atherosclerotic plaques. Rupture-prone vulnerable plaques are known to be characterized by a thin fibrous cap of less than 65 µm, large necrotic cores, high macrophage infiltration, and high inflammatory activity^2–5^. Therefore, to fully characterize plaque vulnerability, it is necessary to evaluate both structural and biochemical information.

In clinical practice, various intravascular imaging devices, such as intravascular ultrasound (IVUS) and intravascular optical coherence tomography (IV-OCT), have been introduced for the diagnosis of atherosclerosis. IVUS is capable of whole volumetric imaging of coronary arteries due to its deep penetration depth, but its low spatial resolution (a few hundred microns) cannot fully assess the microstructure of the plaque^6,7^, including the thickness measurement of the fibrous cap, which is one of the most important features in identifying vulnerable plaques^8–10^. On the other hand, IV-OCT can provide vascular microstructural imaging with a resolution of 10 to 30 microns, about ten times higher than IVUS, allowing accurate measurement of fibrous cap thickness and clear visualization of the thrombus, calcification, and stent struts^11^. Although IVUS and IV-OCT provide morphological information about the internal artery, they have the common limitation of being unable to provide biochemical information about the inflammation and components of the plaques. Therefore, there is an urgent need for a multimodal imaging device that can provide biochemical, compositional, and molecular information about arterial plaques^12^.

Recently, various multimodal catheter-based imaging devices have been developed for comprehensive imaging of atherosclerotic plaques, including IVUS/near-infrared spectroscopy (NIRS)^13,14^, IV-OCT/near-infrared fluorescence (NIRF)^15–18^, and IV-OCT/near-infrared autofluorescence (NIRAF)^19,20^. IVUS/NIRS can characterize the lipid content in atherosclerotic plaques and the arterial structure. However, it cannot detect plaque inflammation, which is highly associated with plaque rupture. IV-OCT/NIRF provides co-localized microstructural and molecular imaging of inflammatory activity^15^, macrophage accumulation^17^, and thrombus formation^21^. However, it requires exogenous contrast agents that target specific molecular information^16,18^, which is slowing its translation into the clinic. Recently, Ughi *et al*.^20^ demonstrated that IV-OCT/NIRAF is a safe and effective intracoronary imaging method in human patients by showing that the autofluorescence signal from an endogenous fluorophore within the plaque is associated with a high-risk plaque phenotype. However, the biological or molecular nature of the autofluorescence signal remains unclear. Also, the strength of the autofluorescence signal is susceptible to errors caused by the changes of environmental conditions, such as laser power fluctuation and sample distance, so IV-OCT/NIRAF requires a complex calibration process^22^.

Fluorescence lifetime imaging (FLIm) is an optical imaging technology that measures the lifetime of fluorescence emissions, a characteristic of fluorophores. During the past decade and more, FLIm has emerged as a diagnostic technology for CAD^23,24^. Because the biochemicals that constitute vascular tissues, such as collagen, elastin, macrophages, and low-density lipoprotein, emit characteristic autofluorescence when irradiated in the ultraviolet region, they can be visualized and distinguished by measuring the lifetime of autofluorescence^25^. However, FLIm cannot provide the structural information needed to interpret the biochemical information, so studies combining FLIm with IVUS^26–29^ or OCT^30^ have been performed. Intravascular imaging of swine coronary arteries *in vivo*^31^ and human coronary arteries *ex vivo*^32^ using an IVUS/FLIm catheter showed that different types of plaque could be clearly distinguished using the structural and biochemical information obtained from IVUS and FLIm. However, clinical translation of that technology is impeded by a slow pullback speed and a relatively large imaging catheter that combines optical fiber and an ultrasound transducer. On the other hand, a combined OCT/FLIm bench-top system has been shown to be able to distinguish plaque types in human coronary arteries *ex vivo*^33^. Recently, a fiber-based imaging platform that combines OCT and FLIm was reported using a gradient-index (GRIN) lens and a double-clad fiber with a cladding diameter of 400 µm^34^. For *in vivo* intravascular imaging, thin optical fiber should be used with helical beam-scanning. An IV-OCT/FLIm catheter system could be rapidly translated into the clinic if it could use an intravascular imaging catheter of the same size and imaging speed as conventional IV-OCT. Some technical challenges have needed to be addressed, including the development of a hybrid optical rotary joint (ORJ)^35^ and multimodal imaging catheter that covers the ultraviolet–visible (UV-VIS) spectrum for FLIm and the near-infrared (NIR) for OCT.

In the present study, we present the development of an IV-OCT/FLIm catheter system that simultaneously visualizes the structure and biochemical composition of blood vessels and plaques. The combined OCT/FLIm system was developed based on our high-speed, precision-enhanced FLIm system and a custom-built swept-source OCT system^36^. The hybrid ORJ was developed using optical fibers of various sizes and ball- and GRIN-lenses to ensure high efficiency over a broad wavelength range. The imaging catheter was built using a double-clad fiber that ensures the simultaneous acquisition of OCT and FLIm at the same position. The outer diameter of the custom-built imaging sheath is 1.04 mm, similar to those already used clinically. The ORJ and imaging catheter was engineered to rotate up to 100 rotations per second. Because the imaging speed and dimension of the imaging catheter are comparable to those of the IV-OCT currently used in clinical practice, we expect that our IV-OCT/FLIm catheter system can be easily translated into clinical use. We present structural/biochemical imaging of the plaque in an atherosclerotic rabbit artery acquired *in vivo* using our newly developed IV-OCT/FLIm catheter system without an exogenous contrast agent.

## Results

### IV-OCT/FLIm system

**Figure 1:**
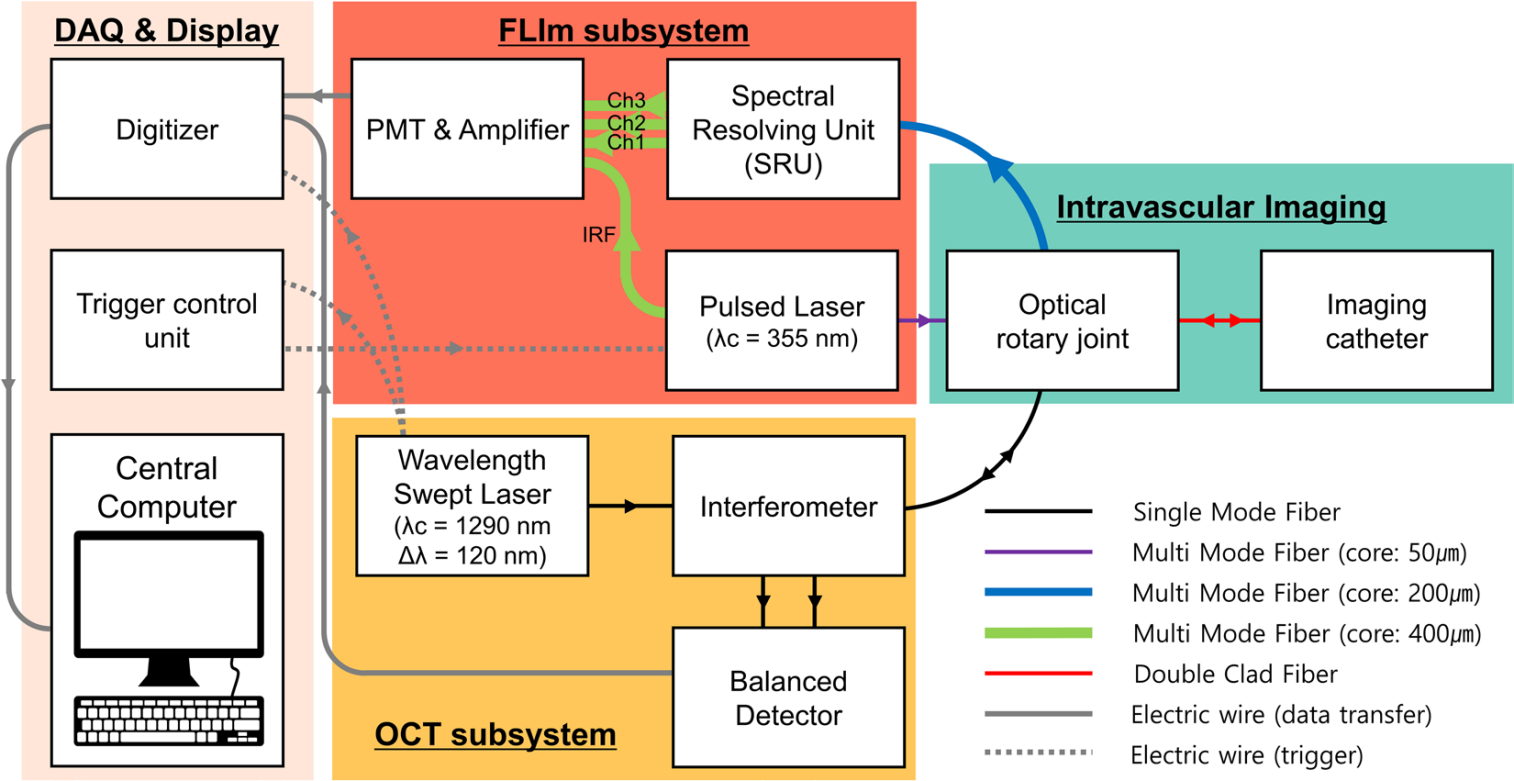
Schematic diagram of the fully integrated IV-OCT/FLIm system for structural and biochemical imaging simultaneously. IV-OCT/FLIm system consisted of OCT subsystem (yellow), FLIm subsystem (red), an intravascular imaging part (green) and data acquisition and display part (light orange). Black, purple, blue, green and red solid lines: optical fiber; gray solid lines: electric wire for transferring data; gray dashed lines: electrical wire for triggering; IRF: instrument response function; Ch1: channel 1; Ch2: channel 2; Ch3: channel 3.

We developed a fully integrated IV-OCT/FLIm system that simultaneously acquires the tomographic structure and fluorescence lifetime of a sample. A schematic diagram of the IV-OCT/FLIm imaging system is shown in Fig. 1. The OCT was developed based on a swept-source laser with a sweeping rate of 120 kHz for high-speed imaging. The sample arm of the OCT was connected with a single-mode fiber (SMF) to a custom-built hybrid ORJ that integrates the OCT and FLIm beams into a single imaging catheter and rotates the catheter for helical scanning. Backscattered light from the sample is detected by a balanced detector through the imaging catheter and the ORJ. 355 nm UV pulses with a repetition rate of 30 kHz are coupled to a multi-mode fiber for FLIm excitation and delivered to the sample through the ORJ and the imaging catheter. Autofluorescence data collected from the sample through the imaging catheter are input to a spectral resolving unit (SRU) and divided into three spectral band channels (Ch1, 390/40 nm; Ch2, 452/45 nm; Ch3, 542/50 nm). For high speed FLIm imaging, we adopted the multi-spectral analog mean-delay (AMD) method^36–39^, which is much less computationally burdensome and much less affected by modal dispersion than the deconvolution method. The fluorescence lifetime is acquired for every four A-lines of the OCT, which is adequate considering that the spatial resolution of the OCT and FLIm is 24 µm and 80 µm, respectively. The details of the development of the OCT/FLIm system were described in our previous study^36^.

### ORJ and imaging catheter for IV-OCT/FLIm

**Figure 2:**
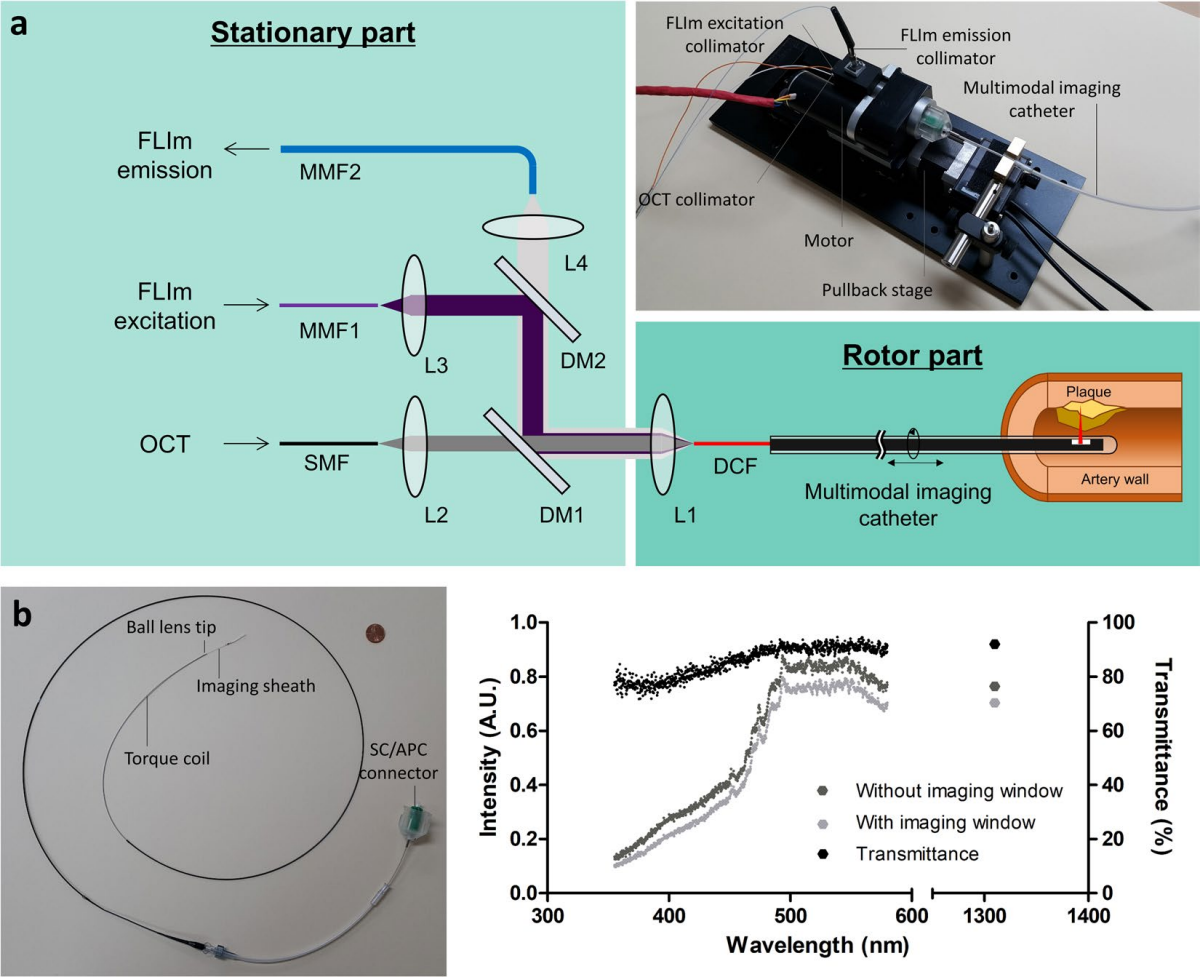
Optical rotary joint (ORJ) and multimodal imaging catheter. (**a**) Schematic diagram and photograph of ORJ. L, lens; DM, dichroic mirror; SMF, single-mode fiber; MMF, multi-mode fiber; DCF, double-clad fiber. (**b**) Photograph of multimodal imaging catheter with a 1 cent coin. The outer diameter of the imaging sheath is 1.04 mm. (**c**) Intensity plot without (dark gray) and with (gray) imaging window and transmittance plot (black) of imaging window. Transmittance of imaging window is more than 75% at OCT and FLIm light.

The ORJ was custom designed to maintain high efficiency over the wide wavelength range (UV–NIR) of OCT and FLIm using multiple fiber couplers with different types of collimators (Fig. 2a). We used a double-clad fiber (DCF) to efficiently combine the OCT and FLIm light in the multimodal imaging catheter. The single-mode core and the multi-mode cladding of the DCF, with diameters of 9 µm and 105 µm, respectively, were used for OCT and FLIm. The low index coating and the protective outer coating has a diameter of 125 µm and 250 µm, respectively, which is exactly the same to SMF-28. We optimized the length between the collimator, L1, and the DCF to maximize OCT light coupling in the NIR. Thus, maintaining high coupling efficiencies for FLIm excitation and emission was challenging due to the chromatic focal shift. To minimize the effects of the chromatic focal shift, the FLIm excitation fiber (MMF1) and emission fiber (MMF2) have core diameters of 50 µm and 200 µm, respectively, which are half and double the diameter of the DCF cladding (Supplementary information and Supplementary Fig. S1). The ORJ maintains good coupling efficiency while rotating, with an insertion loss of −1.3 ± 0.23 dB, −2.3 ± 0.15 dB, and −1.1 ± 0.17 dB for the SMF to DCF, MMF1 to DCF, and DCF to MMF2, respectively.

We fabricated the multimodal imaging catheter, consisting of an imaging core and an imaging sheath, as shown in Fig. 2b. The distal end of the imaging core is made of a ball lens to focus and collect OCT and FLIm light (Supplementary Fig. S2). The proximal end of the imaging catheter is made of an SC/APC connector so that it can be easily attached to and detached from the ORJ. The imaging sheath was custom-made with an outer diameter of 1.04 mm and can be rotated and pulled back up to 100 rps and 40 mm/s, respectively, which are comparable to IV-OCT currently used in clinical practice (100~180 rps and 20~40 mm/s)^40–43^. We used fluorinated ethylene propylene (FEP) tubing as a distal imaging window because it has high transmittance from UV to NIR. As shown in Fig. 2c, the transmission spectra from 355 nm to 570 nm for FLIm and 1310 nm for OCT were measured with (gray dots) and without the imaging window (dark gray dots) to evaluate the transmittance (black dots). The transmittance of the imaging window in OCT and FLIm light was more than 92% and 75%, respectively.

### Validation of OCT and FLIm through phantom experiment

**Figure 3:**
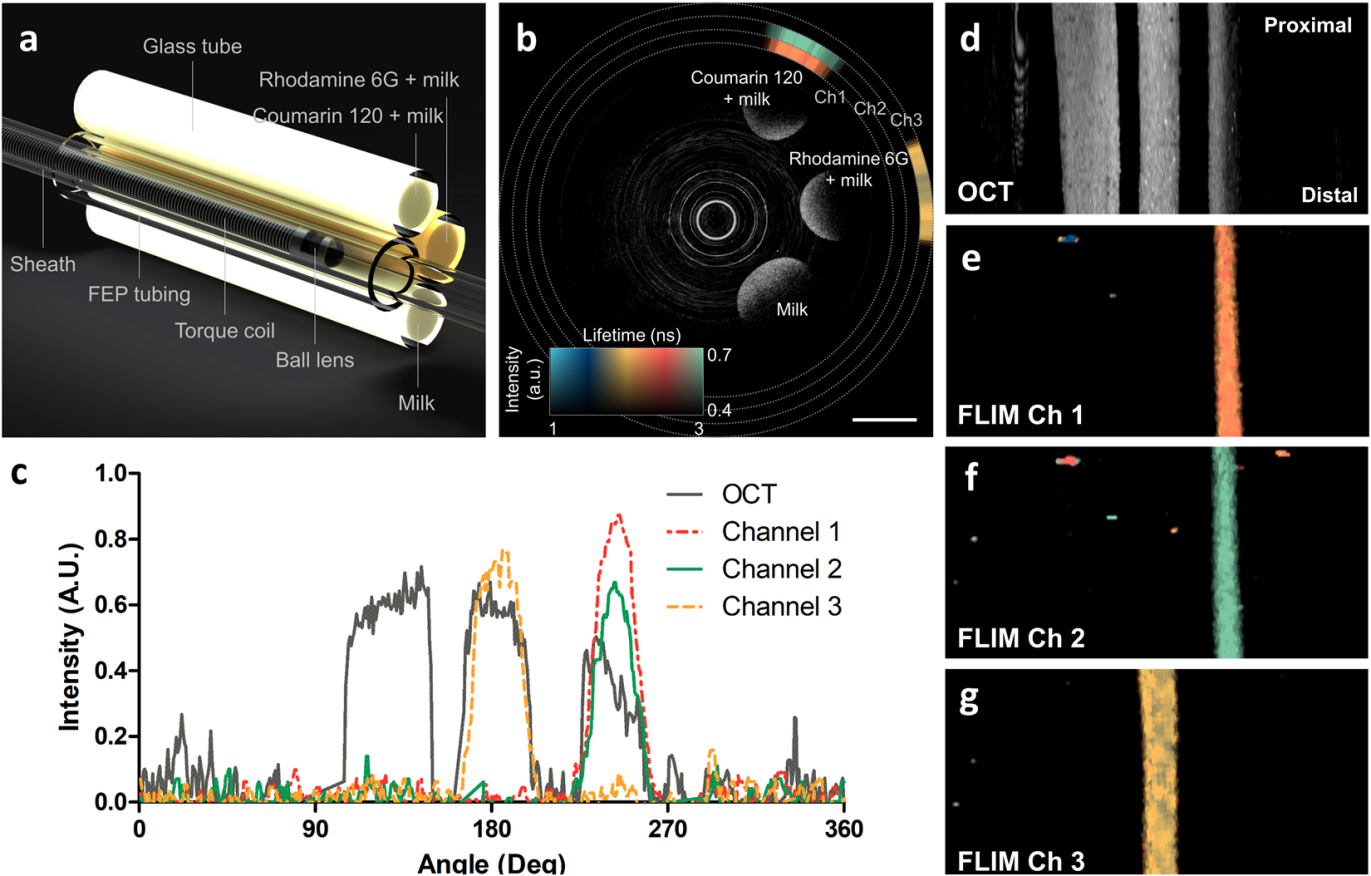
Phantom experiment validating the co-registration between OCT and FLIm. (**a**) Schematic diagram of the phantom consisting of glass tubes filled with milk and standard fluorophores. (**b**) Representative cross-sectional image of phantom. Grayscale and pseudo color represent OCT image and fluorescence lifetime, respectively. (**c**) Intensity graph of OCT and fluorescence along the rotational angle. (**d**) OCT enface map of maximum projection. The two-dimensional fluorescence lifetime maps obtained from channel (**e**) 1, (**f**) 2, and (**g**) 3. Scale bar: 1 mm.

First, we validated the accuracy and precision of the FLIm by measuring multispectral fluorescence lifetimes of standard fluorophores, such as Coumarin 120 and Rhodamine 6 G (Supplementary information). In ethanol, Coumarin 120 and Rhodamine 6 G have emission peaks of 431 nm and 552 nm, respectively. The measured fluorescence lifetimes of Coumarin 120 were 3.61 ± 0.05 ns on channel 1 and 3.59 ± 0.05 ns on channel 2. The measured fluorescence lifetime of Rhodamine 6 G on channel 3 was 3.97 ± 0.05 ns. The difference was less than 1.38% for all channels compared to the values in a literature^44,45^. The precision was only about 0.05 ns without any averaging. Then, to confirm the co-registration between OCT and FLIm, we simultaneously acquired OCT and FLIm images of a phantom consisting of glass tubes filled with milk, milk and Coumarin 120, and milk and Rhodamine 6 G, as shown in Fig. 3a. Figure 3b is a representative cross-sectional image of the phantom. Strong scattering signals from three tubes filled with milk and fluorophores are clearly observable in the grayscale OCT images. The multispectral fluorescence lifetimes are color-coded and intensity-weighted and then displayed as three rings outside the OCT image, since intensities as well as lifetimes of the multispectral autofluorescence emissions contain information on biochemical compositions^27^. The fluorescence lifetime of the milk containing Coumarin 120 was measured on channels 1 and 2 at the corresponding position of the OCT image with good co-registration. The fluorescence lifetime of the milk containing Rhodamine 6 G was measured on channel 3 at the corresponding position of the OCT image with good co-registration. There was no fluorescence signal from the milk. The normalized intensities of OCT and fluorescence along the rotational direction indicate that the two modalities were well co-registered with a spatial offset between the OCT and FLIm images of 0.24+/−0.10 degree (Fig. 3c). This corresponds to 6.13+/−2.66 μm, assuming a diameter of 3 mm. This spatial offset is lower than the spatial resolution of OCT and FLIm; thus the spatial co-registration is excellent. Given the good co-registration between OCT and FLIm, the OCT enface map and two-dimensional fluorescence lifetime maps show multimodal images of the sample at a glance (Fig. 3d–g).

### *In vivo* OCT/FLIm imaging in rabbit aorta

**Figure 4:**
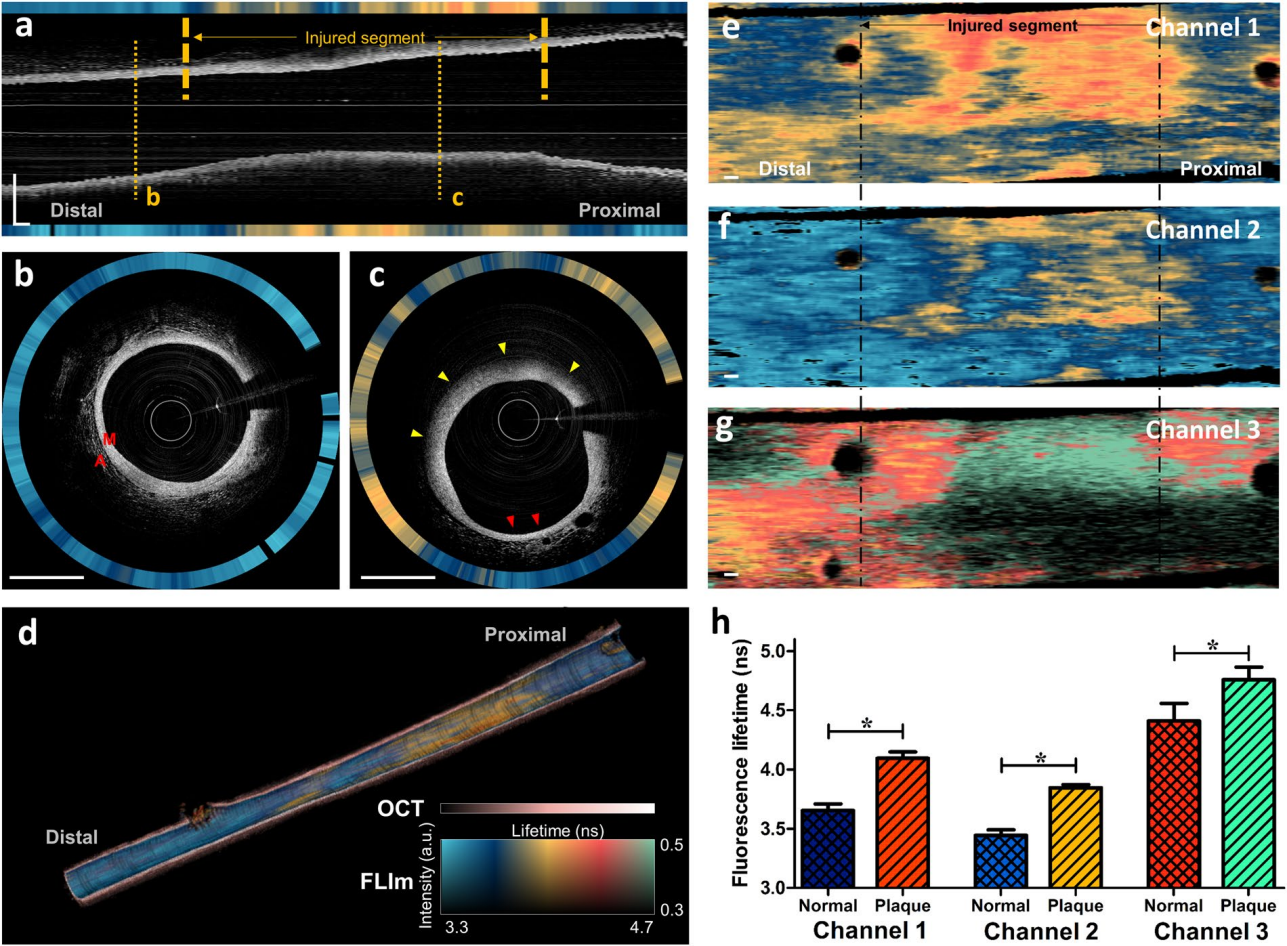
*In vivo* OCT/FLIm imaging results in a rabbit aorta. (**a**) Longitudinal OCT/FLIm images of a rabbit aorta. Grayscale and pseudo color represent the OCT image and fluorescence lifetime, respectively. (**b**) Representative cross-sectional image of normal aorta. Structures of normal aorta, including media (M) and adventitia (A) are clearly visible. (**c**) Representative cross-sectional image of atherosclerotic plaque. It shows lipid-rich plaque with thickened media and diffuse borer (yellow arrowheads) suggesting presence of lipid in OCT, and it also contains normal area (red arrowheads). (**d**) 3D rendering longitudinal cut-view image. The fluorescence lifetime is overlaid on the luminal surface of the OCT 3D rendering. The fluorescence lifetime in (**a**–**d**) was measured on channel 2. The 2D fluorescence lifetime maps measured on channel (**e**) 1, (**f**) 2, and (**g**) 3. The horizontal and vertical directions are the pullback direction and rotational direction, respectively. (**h**) Comparison of the averaged fluorescence lifetime between the normal aorta and atherosclerotic plaque. Results are presented as means ± standard deviation. *P < 0.001, by unpaired t-test. Scale bars: 1 mm. M, media; A, adventitia.

To demonstrate our system’s clinical feasibility for characterizing structural and biochemical features of atherosclerotic plaque, we simultaneously acquired OCT/FLIm images of lipid-rich plaques and a normal aorta in a rabbit model using our IV-OCT/FLIm catheter system *in vivo* (Supplementary Video 1). Figure 4 shows representative IV-OCT/FLIm images from the rabbit aorta. The longitudinal OCT image clearly shows thickening of the aortic wall caused by the formation of atherosclerotic plaque in the injured segment. The simultaneously obtained fluorescence lifetime in channel 2 is much longer in the balloon-injured segment than in the non-injured segment (Fig. 4a). In the cross-sectional OCT image of the non-injured segment (Fig. 4b), a thin media layer is visible without intimal thickening, and connective tissue is also visible in the adventitia, showing morphological features typical of a normal aorta^46^. The corresponding fluorescence lifetime in channel 2 is homogeneous. On the other hand, the cross-sectional OCT image of the injured segment (Fig. 4c) shows detailed structural features of the atherosclerotic plaque, such as loss of layered intima, media and adventitia structure, significant intimal thickening, and signal attenuation caused by lipids^47^. In the atherosclerotic plaque (yellow arrowheads in Fig. 4c), the fluorescence lifetime in channel 2 is significantly longer than in the normal aorta. Interestingly, there is also a normal-looking portion (red arrowheads in Fig. 4c) in which the morphological features and fluorescence lifetime are similar to those in the non-injured segment (Fig. 4b). The morphological characteristics and fluorescence lifetime distribution described above can also be seen at a glance in the 3-dimensional rendering of the longitudinal cut-view image (Fig. 4d). We only showed fluorescence lifetime in channel 2 in Fig. 4a–d, since it is more straightforward to visualize one channel than to display all channels at once. Among the three channels, channel 2 was demonstrated, because the spectral band of channel 2 overlaps the fluorescence spectra of various biochemical compositions, such as elastin, collagen, NADH, and LDL^25,48^. The fluorescence lifetime distributions acquired from all channels are represented in 2-dimensional (2D) fluorescence lifetime maps (Fig. 4e–g). In all channels, longer fluorescence lifetimes were observed in the injured segment that had the atherosclerosis lesion. The difference in fluorescence lifetime between the normal aorta and the atherosclerotic plaque was statistically significant (Fig. 4h, Ch1, 3.65 ± 0.06 ns vs. 4.10 ± 0.05 ns, p < 0.0001; Ch2, 3.44 ± 0.05 ns vs. 3.85 ± 0.03, p < 0.0001; Ch3, 4.41 ± 0.15 ns vs. 4.76 ± 0.11 ns, p < 0.0001).

### Histopathological validation

**Figure 5:**
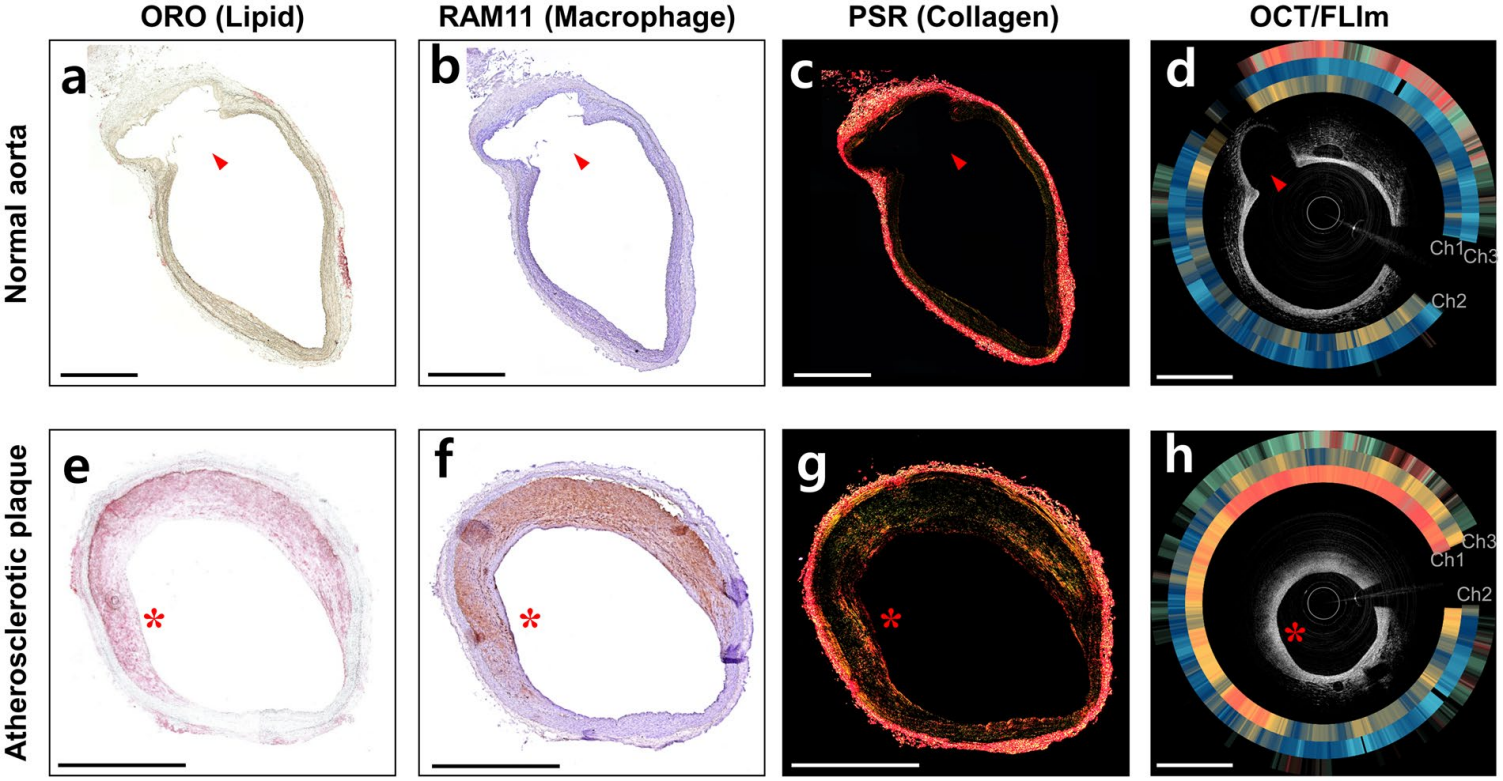
Histopathological staining and cross-sectional OCT/FLIm images of (**a**–**d**) normal aorta and (**e**–**h**) atherosclerotic plaque. (a, e) Oil red O (ORO) stained tissue that represents lipids (red color). (**b**,**f**) Immunohistochemistry with RAM 11 antibody (brown color) that represents macrophages. (**c**,**g**) Picrosirius red (PSR) stained tissue that represents collagen types I (yellow-orange birefringence) and III (green birefringence). (**d**,**h**) Cross-sectional OCT/FLIm images of rabbit aorta. Intensity-weighted fluorescence lifetime is expressed as rings from channels 1, 2 and 3 (in turn from inner to outer) outside the OCT images. Morphological landmarks, such as side branch (red arrows) and plaque shape (red asterisks), show the match between the histopathological sections and the *in vivo* OCT/FLIm cross-sectional images. Fluorescence lifetime colormap is the same as given in Fig. 4. Scale bars: 1 mm.

After IV-OCT/FLIm imaging, we performed histopathological analyses to confirm the *in vivo* results and correlate the fluorescence lifetime changes with the tissue compositional changes. The Oil red O (ORO) (Fig. 5a) and RAM-11 (Fig. 5b) stained sections of the normal aorta show that the normal aorta rarely contains lipid and macrophages, respectively. Also, the normal aorta consists mainly of collagen and elastin, which is distributed in the internal elastic lamina and in the elastic lamellae of aortic media^49^, as shown in the picrosirious red (PSR) stained section (Fig. 5c). On the other hand, atherosclerotic plaque is characterized by lipid accumulation, active inflammation, macrophage infiltration and lipid-laden foam cell formation, and increased deposition of collagen type I^50^. In our rabbit model of atherosclerosis, induced by balloon denudation and high-cholesterol diet, this can be confirmed by abundant lipids, macrophages, and collagen type I in ORO (Fig. 5e), RAM-11 (Fig. 5f) and PSR (Fig. 5g) stained sections, respectively (red asterisks in Fig. 5e–f).

Distinct fluorescence lifetime changes are observed in the *in vivo* IV-OCT/FLIm imaging (Fig. 5d,h). The fluorescence lifetime on channel 3 is longer in the atherosclerotic plaque (Fig. 5h) than in the normal aorta (Fig. 5d), due to the substantial lipid accumulation on the atherosclerotic plaque as shown in the ORO staining section (Fig. 5e), as the plaques containing high levels of lipids increase fluorescence lifetime in channel 3^25^. In channels 1 and 2, where the spectral bands overlap the emission spectra of elastin and collagen, longer fluorescence lifetimes were obtained in the atherosclerotic plaque (Fig. 5h) than in the normal aorta (Fig. 5d). In the normal aorta, which is a type of elastic arteries, elastin might contribute the uniform and relatively short fluorescence lifetime^25^. In the plaque, increased deposition of collagen type I, which has a longer fluorescence lifetime than collagen type III^51^, during plaque formation contributes to the prolonged fluorescence lifetime, as confirmed by the increase in yellow-orange birefringence in the picrosirius red (PSR) staining of the atherosclerotic plaque (Fig. 5g) compared to the normal aorta (Fig. 5c)^52,53^. An increase in macrophages or foam cells in the atherosclerotic plaque might be another factor that prolongs the fluorescence lifetime in channel 2^54^, where the spectral band overlaps the emission spectra of nicotinamide adenine dinucleotide (NADH)^48^ and low-density lipoprotein (LDL)^25^, which can be bonded to and ingested by macrophages, respectively. The RAM 11-immunostained section of the atherosclerotic plaque (Fig. 5f) shows a dense accumulation of macrophages over the tissue, whereas macrophages were barely detected in the normal aorta (Fig. 5b). The comparison results between the histological analysis and the *in vivo* IV-OCT/FLIm images show that the multispectral lifetime changes are closely related to the histopathological changes of the arterial tissue, including macrophage infiltration, lipid deposition, and collagen synthesis and degradation. Thus, the multispectral FLIm can be used to precisely analyze tissue compositional changes associated with atherosclerosis.

## Discussion

FLIm, combined with morphological imaging methods such as IVUS or OCT, is useful for characterizing and classifying atherosclerotic plaques in a label-free manner^26,30^. In particular, FLIm and OCT are expected to combine efficiently because they are both optical imaging methods. In fact, FLIm and OCT have been combined in a bench-top setup to demonstrate comprehensive imaging of the coronary artery^33^, and an endoscopic multimodal imaging platform was recently developed using a double-clad fiber^34^. However, IV-OCT/FLIm catheter systems ready for preclinical and clinical studies of comprehensive, *in vivo* intravascular artery imaging have not yet been reported, partly due to the difficulty of manufacturing a hybrid ORJ and an imaging catheter with high coupling efficiency over a wide spectral range (UV to NIR). In this study, we have presented a hybrid ORJ that efficiently combines FLIm and OCT into a single dual-modal imaging catheter using a double-clad fiber. The wide spectral range required for FLIm and OCT (from UV to visible and NIR, respectively) leads to a chromatic focal shift that makes maintaining high coupling efficiency difficult. To increase the coupling efficiency for both FLIm and OCT, we used multiple fibers with different diameters. We also adopted the AMD FLIm method that broadens the measured fluorescence pulses through low-pass filtering, so that the fluorescence lifetime can be measured with high precision even at low sampling rates^36,37^. The AMD FLIm method is suitable for *in vivo* imaging, because it enables fast FLIm acquisition with high photon efficiency and is relatively less affected by modal dispersion than other methods. We then successfully performed IV-OCT/FLIm imaging to provide structural and biochemical information about a rabbit aorta *in vivo*. To promote rapid translation into clinical use for our IV-OCT/FLIm catheter system, we used a DCF with the same physical dimensions as the SMF already used in clinical IV-OCT catheters. The DCF with the same core, cladding, and outer coating as the SMF28, has an additional inner cladding with a diameter of 105 µm. This inner cladding is used for FLIm excitation and emission, and the core is used for OCT. Because the outer cladding, with a diameter of 125 µm, is identical to the SMF28, the multimodal imaging probe could be fabricated using the same procedure used for IV-OCT^55^.

When combining the OCT and FLIm beams into the DCF imaging catheter, we optimized the fiber diameters and collimator distances to maximize the coupling efficiency of the hybrid ORJ. First, the length between the OCT collimator, L1, and the DCF was optimized for OCT light coupling (Fig. 2a). Then we calculated the insertion loss of the FLIm excitation and emission as a function of the core diameters of MMF1 and MMF2, respectively, as shown in Supplementary Fig. S1, using ZEMAX. When the core diameters of MMF1 and MMF2 are smaller and larger than 50 μm and 200 μm, respectively, the insertion loss for excitation and emission approached the minimum. Considering the modal dispersion and the bending radius of the multi-mode fiber, we chose MMF1 and MMF2 with core diameters of 50 μm and 200 μm, respectively. Then, we adjusted the length between the excitation collimator, L3, and MMF1 to maximize the excitation efficiency. Finally, the length between the emission collimator, L4, and MMF2 was adjusted to maximize the emission efficiency. Thus, we were able to maintain an insertion loss for OCT (−1.3 dB) and FLIm emission (−1.1 dB) suitable for imaging. Although the insertion loss for UV excitation is still not negligible (−2.3 dB) and the absorption by the fiber itself causes additional loss when illuminating the sample, the excitation power loss can easily be compensated by increasing the laser power. The resultant excitation power on the sample needed to acquire the FLIm signal is much lower than the maximum permissible exposure in the American National Standards Institute (ANSI) standard.

Because we used a UV pulsed laser as an excitation source for FLIm, the background noise, such as autofluorescence from the optical fiber itself, could affect the FLIm measurements. In fact, the germanium-doped core and low refractive index cladding of the DCF strongly absorb UV light and produce autofluorescence under UV excitation^56,57^. Because its spectrum overlaps the autofluorescence spectrum of the arterial tissue, the autofluorescence of the optical fiber should be minimized and removed for accurate FLIm measurements. Therefore, the UV excitation light was guided through the cladding of the DCF instead of the core. However, when the UV excitation light is coupled to the cladding of the DCF, a portion of the UV light is inevitably coupled to the core of the DCF, resulting in unwanted autofluorescence artifacts. Fortunately, there was time delay between the autofluorescence of the DCF and the autofluorescence of the artery because the autofluorescence of the DCF was emitted mainly from the proximal end of the fiber, where the UV light was coupled to the DCF, whereas the autofluorescence of the artery travels through the 1.6-meter-long imaging catheter. Therefore, the autofluorescence of the DCF was sequentially separated from the tissue autofluorescence and removed from further calculation (Supplementary Fig. S3). Additionally, the use of a contrast agent for blood flushing in OCT/FLIm imaging was problematic due to the high UV absorption of the agent. In our *in vivo* IV-OCT/FLIm imaging of rabbit aorta, saline flushing has been successfully used because of its very low absorption of UV light. For clinical applications in humans, high-viscosity flushing agents, such as dextran 40, which has been reported as a promising candidate for OCT/FLIm flushing medium^58^, need to be tested for intracoronary OCT/FLIm imaging. This is because high-viscosity flushing agents effectively remove blood from the imaging field without loss of OCT image quality with a small amount of use^59^, thereby enabling safe clinical application to humans.

In the case of intravascular optical imaging, it is generally known that as the distance between the catheter and the sample increases, the collection efficiency and the intensity of the detected signal decreases. Therefore, additional post-processing is required to compensate for that distance effect^15,16,22^. Our IV-OCT/FLIm catheter has the same effect, i.e., the fluorescence intensity decreases as the distance to the sample increases. However, because the fluorescence lifetime is independent of the fluorescence intensity, FLIm has the advantage of excluding any misinterpretation caused by variations in the fluorescence intensity^60,61^. We confirmed that our IV-OCT/FLIm catheter can precisely measure the fluorescence lifetime without being influenced by the fluorescence intensity (Supplementary Fig. S4).

Because the penetration depth of UV light is about 200 µm^62^, FLIm provides biochemical information of the arterial wall only near the luminal surface, where the biocompositional changes significantly affect the vulnerability of the plaque. Phipps *et al*. investigated the collagen to lipid ratio in fibrous cap of human plaque to assess structural integrity of the fibrous cap^63^. Fatakdawala *et al*. used FLIm to distinguish between relatively stable thick-cap fibroatheromas and rupture-prone Thin-cap fibroatheromas^32^. Therefore, the specific information near the luminal surface due to the shallow penetration depth of FLIm could be rather useful for assessing the pathophysiology of high-risk plaques.

In this study, atherosclerosis rabbit model was established by high cholesterol diet and balloon injury, and IV-OCT/FLIm imaging *in vivo* was performed for the structural/biochemical characterization of the atherosclerotic plaque. The average diameters of the rabbit aorta measured by OCT and angiograph images were 3.17 ± 0.49 mm and 3.15 ± 0.32 mm, respectively, similar to the size of human coronary arteries^64,65^, showing the clinical validity of the experiment^58,66^. Normal rabbit aorta is a type of elastic artery mainly composed of collagen and elastin^67,68^. In contrast, atherosclerotic lesions in the rabbit aorta are characterized by increase in lipid accumulation, infiltration of macrophages, and formation of collagen type I^50^, as shown in our results. Biochemical imaging with our IV-OCT/FLIm catheter system showed longer fluorescence lifetimes in all three channels in atherosclerotic plaques with increased lipid, macrophages, and collagen type I than in normal aorta with elastin and collagen type III. This result is consistent with a previous work that reports plaques with high collagen or high lipid exhibited significantly longer fluorescence lifetime than arterial tissues with low collagen/low lipid^25^. Longer fluorescence lifetimes in plaque might be due to the longer fluorescence lifetime of collagen type I than that of elastin and collagen type III^51^. Also, macrophage infiltration, foam cell formation, and increased lipid content have been reported to prolong the fluorescence lifetime^24,69,70^. Coronary artery, a type of muscular arteries, might show different fluorescence lifetimes from the rabbit aorta, a type of elastic arteries, used in this study. Also, the plaque phenotype in human patients is very different from the plaque induced by the injury and hypercholesterolemic diet in our rabbit model of atherosclerosis. Thus, further studies on plaques in human coronary arteries should be conducted to analyze the meaning of the FLIm signals. Fluorescence lifetime changes and fluorescence intensities in multiple spectral ranges are associated with complex biochemical processes, including collagen synthesis and degradation, elastic filaments, macrophage infiltration, and increased lipid content. Thus, machine learning algorithms could be useful for analyzing FLIm signals to accurately identify the biochemical composition of a plaque^63^. Also, microscopic features of OCT can be included in the analysis to determine pathological changes in the arteries and assess plaque vulnerability^2–5^.

In conclusion, we have developed an IV-OCT/FLIm catheter system that simultaneously provides both morphological and biochemical information. Then, we successfully acquired microstructure and fluorescence lifetime data about atherosclerotic plaques in a rabbit aorta *in vivo* and demonstrated distinct fluorescence lifetime changes in the plaque compared with the normal aorta. This novel, comprehensive imaging catheter can be a promising tool for preclinical and clinical studies by differentiating plaque types, analyzing inflammation, and assessing plaque vulnerability. In particular, rapid translation into clinical use is expected because no exogenous contrast agent is required, and the imaging procedures and physical properties of the multimodal imaging catheter are the same as those of IV-OCT already used clinically.

## Methods

### IV-OCT/FLIm system

We developed the IV-OCT/FLIm system as shown in Fig. 1. The OCT was built based on a 120 kHz swept-source laser with a center wavelength and bandwidth of 1290 nm and 120 nm, respectively. The average power on the sample was 30 mW. The OCT interference signal was detected by a dual-balanced detector (HCA-S, Femto, Berlin, Germany). The FLIm used a UV (355 nm) pulsed laser with a repetition rate of 30 kHz, a pulse width of 1.56 ns, and a pulse energy of 3.5μJ. The spectral ranges of each channel of the SRU were as follows: channel 1, 370–410 nm; channel 2, 430–475 nm; channel 3, 517–567 nm. The instrument response function and autofluorescence of each channel were sequentially measured at time intervals of 72 ns using optical delay lines with fiber lengths of 1 m, 16 m, 31 m, and 46 m. An amplifier with a bandwidth of 150 MHz was used to broaden the pulse width for calculating fluorescence lifetime using AMD method^36,37^. The OCT swept laser also generated a trigger signal at the beginning of each wavelength sweep. This 120 kHz trigger signal was used for a 2-Ch 14-bit high-speed digitizer (PX1440A, Signatec, CA, USA) that acquired the OCT interference signal in one channel and the FLIm signal in the other channel at a rate of 340 MHz per channel. Also, the trigger signal was connected to the counter of a DAQ board (USB-6434, National instruments, TX, USA) that generated another trigger signal after every four input triggers, resulting in a repetition rate of 30 kHz for the FLIm pulsed laser.

### ORJ of the IV-OCT/FLIm

We designed the hybrid ORJ, which combines OCT light, FLIm excitation, and FLIm emission between the stationary optical system and the rotary imaging catheter, using multiple collimators, dichroic mirrors, and filters. Thus, the ORJ consists of a stationary part (bright green) and a rotor part (dark green) as shown in the schematic diagram and photograph in Fig. 2a. The OCT illumination light and FLIm excitation light propagate through the SMF (SMF-28e, Corning, NY, USA) and MMF1 (FG050UGA, THORLABS, NJ, USA), respectively. Collimating lenses L2 (f = 1.9 mm, GRIN lens) and L3 (f = 3.3 mm, fused silica plano-convex lens) of the stationary part collimate the OCT illumination light and FLIm excitation light, respectively. Then, the two beams are combined using a dichroic mirror, DM1 (FF665-Di02, Semrock, NY, USA). Collimating lens L1 (f = 2.5 mm, fused silica ball lens) of the rotor part couple the OCT illumination light and FLIm excitation light to the DCF (SM-9/105/125-20 A, NUFERN, CT, USA) core and cladding, respectively. The FLIm excitation and emission light are divided by a dichroic mirror, DM2 (FF376-Di01, Semrock, NY, USA). Collimating lens L4 (f = 3.2 mm, fused silica half-ball lens) couple FLIm emission light to MMF2 (FG200UCC, THORLABS, NJ, USA). To reduce the optical loss, the lenses are anti-reflection coated.

### Multimodal imaging catheter

The multimodal imaging core was manufactured using DCF (SM-9/105/125-20 A, NUFERN, CT, USA) to enable simultaneous OCT and FLIm imaging. The DCF was spliced to coreless fiber (MM125, FIBERCORE, Southampton, UK) that was processed into a ball shape using a fusion splicer (GPX-3300, THORLABS, NJ, USA). To enable side-viewing, the ball-shaped coreless fiber was polished at 41 degrees^55^. The imaging sheath was constructed by replacing the imaging window of the IVUS sheath (Atlantis SR pro, Boston Scientific, MA, USA) with FEP tubing with an inner diameter of 0.74 mm and an outer diameter of 1.04 mm (AWG21, Zeus, SC, USA). The spectra were measured using a xenon lamp and a spectrometer (USB4000, OceanOptics, FL, USA) in the wavelength range of light used by FLIm with and without the imaging window. At 1310 nm, power was measured using a power meter (PM100D, THORLABS, NJ, USA) and a photodiode power sensor (S132C, THORLABS, NJ, USA) with and without the imaging window. The transmittance of the sheath was calculated by dividing the spectrum and power with the imaging window by those without the imaging window.

### Data recording, real-time display, and image processing

We developed a C++-based graphical user interface for data acquisition, IV-OCT/FLIm imaging catheter system control, and real-time display. The details of data recording and real-time display were described in our previous study^36^. All the images were processed using a C++-based algorithm, ImageJ (version 1.49, US National Institutes of Health, MD, USA), and MATLAB R2015b (The MathWorks, Natick, MA, USA). 1024 OCT A-lines were processed to visualize the cross-section of the phantom in Cartesian coordinates. Corresponding FLIm signals were displayed as three rings outside the OCT image. The acquired fluorescence lifetime was displayed in HSV (hue, saturation and value) pseudo color (intensity weighted lifetime), where the hue and value represent fluorescence lifetime and intensity, respectively. To process three-dimensional rendering images, OCT images covered with the two-dimensional fluorescence lifetime map along the aortic wall were stacked along the pullback direction. Three-dimensional rendering was conducted using OsiriX (The OsiriX Foundation, Geneva, Switzerland). Maximum projection was used to obtain an OCT enface map of the phantom. Fluorescence lifetime 2D maps of the three channels were also generated to compare the co-registration of OCT and FLIm.

### Phantom experiments

We prepared a phantom of glass tubes (OD/ID: 1.5/1.1 mm) filled with milk, milk and Coumarin 120, and milk and Rhodamine 6 G. Milk was selected as the scattering material for OCT. We chose Coumarin 120 (Sigma-Aldrich, MO, USA) and Rhodamine 6 G (Sigma-Aldrich, MO, USA), which have emission peaks in ethanol at 431 nm and 552 nm, respectively, as the standard fluorophores because the emission spectra were suitable for FLIm imaging. The concentrations of Coumarin 120 and Rhodamine 5 G were 100 nM and 10 μM, respectively, which were determined to have fluorescence intensities similar to the autofluorescence of human tissues. The glass tubes were sealed with epoxy and glued to a cylindrical surface of FEP tubing (OD/ID: 1.65/1.35 mm, Zeus, SC, USA). The phantom was immersed in water and helically scanned (50 rps, 20 mm/s) to acquire OCT and FLIm images. The offsets between the OCT and FLIm channels were calculated by comparing the centers of the OCT and fluorescence intensity profiles. The centers of each signal were determined as the center pixels of 1/e-squared width of the maximum values.

### Rabbit model of inflamed lipid-rich plaque

To generate a rabbit model of atherosclerosis, a New Zealand white rabbit (NZWR, DooYeol Biotech, Korea) was balloon injured on the infrarenal aorta at the vertebral L2-L4 position confirmed by angiography and fed a high cholesterol diet. Balloon endothelial denudation was conducted by three pullbacks at a balloon pressure (0.05–0.2 mL). For a week before and three weeks after balloon endothelial denudation, the rabbit was fed a 1% high cholesterol diet (1% cholesterol and 5% peanut oil, C-30293, Research Diets). For seven weeks after the 1% high cholesterol diet, the rabbit received a 0.1% high cholesterol diet (0.1% cholesterol and 5% peanut oil, C-30293, Research Diets).

### *In vivo* OCT/FLIm imaging in rabbit aorta

Intravascular *in vivo* imaging was performed ten weeks after balloon endothelial denudation, to demonstrate the feasibility of IV-OCT/FLIm imaging *in vivo*. Anesthesia was induced with intravenous injection of alfaxalone (Alfaxan; Jurox, Australia) at 3 mg/kg, and maintained with isoflurane (2–3%; JW Pharma, Korea) in oxygen by face mask. A 5-F sheath catheter was inserted into the left carotid artery under anesthesia, and a standard 0.014-inch guide wire was placed in the injured aorta through the sheath catheter. Then, the IV-OCT/FLIm imaging catheter was advanced through the guide wire. OCT/FLIm imaging was performed with a rotation speed of 50 rps, pullback speed of 10 mm/s, and pullback length of 40 mm. In this experiment, we used one rabbit aorta containing both normal and atherosclerotic aorta (~20 mm). The UV fluence rate (1.15mJ/cm2 on aortic wall), is much lower than the ANSI standard’s maximum permissible exposure for skin (3.52mJ/cm2)^28^. Blood was flushed with saline (5 ml/s for 4 sec) to prevent light absorption by blood during the acquisition of the OCT/FLIm images. After *in vivo* OCT/FLIm imaging, the rabbit was euthanized, and the iliac artery and aorta were harvested for histopathological validation. Institutional Animal Care and Use Committee of Korea University (KOREA-2017-0003) approved all the animal experiments, and all animal experiments procedures were performed in accordance with the relevant guidelines and regulations. Statistical analysis of the fluorescence lifetime was conducted in frame unit. First, we separated the atherosclerotic plaque and the normal aorta and manually selected 85 frames of the plaque and 56 frames of the normal area using only OCT images. Then, average fluorescence lifetimes were calculated per frame. Values were expressed as mean and standard deviation for the all three spectral channels. The differences in the fluorescence lifetimes between the atherosclerotic plaque and normal aorta were statistically verified in an unpaired t-test using Prism (GraphPad Software, version 5.0, CA, USA).

### Histopathological validation

After IV-OCT/FLIm imaging, the atherosclerotic segments in the abdominal aorta and iliac arteries were dissected from the euthanized rabbit, preserving the branching arteries, so that these branches could serve as anatomical landmarks for co-registration between *in vivo* OCT cross-sectional and histological images. The dissected arteries were embedded in optimal cutting temperature compound (Tissue-Tek, Sakura Finetek, Japan), immediately snap-frozen, and serially cryosectioned in 10-µm thicknesses. The co-registration of *in vivo* OCT and histological images was performed and assured with reference to the branch vessels, location and plaque morphology and distribution within the imaged aorta. These sections were stained with PSR (Scytek Laboratories, Loggan, UT, USA), and ORO (Scytek Lab., Logan, UT, USA) to determine the collagen and lipid contents in the imaged atherosclerotic lesions, respectively. Immunohistochemical analysis was performed on adjacent frozen section using primary antibody to macrophage (Anti-Rabbit Macrophage Clone RAM 11, Dako, Glostrup, Denmark) at a dilution of 1:500 followed by Dako Envision anti-mouse antibody. Color was developed using the peroxidase substrate diaminobenzidine (Dako, Glostrup, Denmark), and slides were counterstained with Mayer hematoxylin. The histopathological sections have been matched according to the pullback positions. Also, morphological landmarks, such as side branch and lipid-rich plaque, were used to confirm the exact match with the *in vivo* OCT/FLIm images.

## Electronic supplementary material

Supplementary information

Supplementary Video 1
