# Supplementary material for: Comprehensive intravascular imaging of atherosclerotic plaque in vivo using optical coherence tomography and fluorescence lifetime imaging
Source: Sci Rep. 2018 Sep 28;8:14561. doi: 10.1038/s41598-018-32951-9 (PMC6162321; doi:10.1038/s41598-018-32951-9)
Supplement: Supplementary file 1 — Supplementary information [file 41598_2018_32951_MOESM1_ESM.docx]

**SUPPLEMENTARY INFORMATION**

**Comprehensive intravascular imaging of atherosclerotic plaque *in vivo* using optical coherence tomography and fluorescence lifetime imaging**

Min Woo Lee^1, +^, Joon Woo Song^2, +^, Woo Jae Kang^3, +^, Hyeong Soo Nam^1^, Tae Shik Kim^3^, Sunwon Kim^2, 4^, Wang-Yuhl Oh^3, *^, Jin Won Kim^2, *^, and Hongki Yoo^1, *^

^1^Department of Biomedical Engineering, Hanyang University, Seoul, 04763, Republic of Korea

^2^Multimodal Imaging and Theranostic Lab, Cardiovascular Center, Korea University Guro Hospital, Seoul, 08308, Republic of Korea

^3^Department of Mechanical Engineering, KAIST, Daejeon, 34141, Republic of Korea

^4^Department of Cardiology, Korea University Ansan Hospital, Ansan, 15355, Republic of Korea

^+^These authors contributed equally to this work.

^*^These authors shared senior authorship.

Correspondence and requests for materials should be addressed to W.Y. Oh (email: woh1@kaist.ac.kr), J.W. Kim (email: kjwmm@korea.ac.kr), or H. Yoo (email: hyoo@hanyang.ac.kr)

**Optimal core diameters of multi-mode fibers**

In the optical rotary joint of the IV-OCT/FLIm catheter system, chromatic aberration makes it difficult to maintain high coupling efficiency due to the broad spectrum (UV-VIS-NIR) required. If the core diameter of the optical fiber is sufficiently large to cover the increased spot due to the chromatic aberration, high coupling efficiency can be maintained. Thus, to find the optimal core diameters for the optical fibers, we calculated the insertion losses using ZEMAX optical simulation software. We first calculated the UV excitation loss between MMF1 cores of various diameters and a DCF cladding of 105 µm diameter. The wavelength was 355 nm, and the distance between the two lenses, L3 and L1, was 40 mm. Because the core of MMF1 and the DCF cladding can be regarded as an extended source and an extended detector, respectively, the insertion loss of the fiber was calculated using the weighted sum of the insertion loss calculated for various fields within a given radius. We defined three and five field points at the same interval on the MMF1 and DCF. The weights for the fields were determined by assuming that the beam distribution is Gaussian. Similarly, we calculated the fluorescence emission loss between the DCF cladding (105 µm diameter) and MMF2 cores of various diameters. The wavelength was 452 nm, and the distance between the two lenses, L1 and L4, was 50 mm. When the core diameters of MMF1 and MMF2 were smaller than 50 µm and larger than 200 μm, respectively, the excitation and emission insertion loss approached the minimum (Supplementary Fig.S1). Considering the modal dispersion and the bending radius of the multi-mode fiber, we therefore chose MMF1 and MM2 with core diameters of 50 μm and 200 μm, respectively.

**
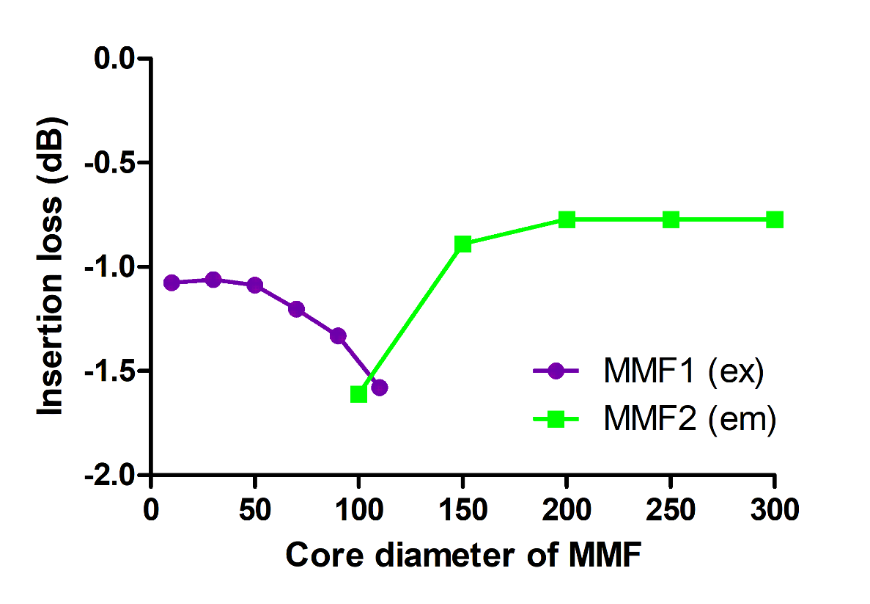
**

Supplementary Figure S1. Insertion loss plot of FLIm excitation and emission as a function of the core diameter of MMF1 and MMF2. When the core diameter of MMF1 (purple) and MMF2 (green) was smaller than 50 µm and larger than 200 μm, respectively, the insertion loss approached the minimum. The insertion losses were calculated using the ZEMAX optical simulation software.

**Distal optics of the imaging core**

In the distal optics of the imaging core, the beam path of the FLIm excitation beam is slightly different from the OCT beam as shown in Supplementary Fig.S2. The OCT beam from the core of the DCF passes through the coreless fiber (red dotted line). Then, it is reflected by the polished surface at a 41 degree and then focused by the ball lens surface. On the other hand, a portion of the FLIm excitation beam from 105-µm multi-mode cladding (blue dotted line) is guided in the coreless fiber unlike the OCT beam (red dotted line), which travels inside the coreless fiber. Additionally, the aperture size of the ball lens is not sufficient for the FLIm excitation beam from the 1st cladding with large diameter and high numerical aperture. Thus, a portion of the FLIm excitation beam will be blocked by the ball lens. Also, the focal length of the FLIm excitation beam is 300 µm shorter than the focal length of the OCT beam, because the wavelength of the FLIm excitation beam is much shorter than that of the OCT beam. Therefore, the magnification of the FLIm excitation beam is less than that of the OCT beam. The measured spot size of the OCT and the FLIm excitation was 24 µm and 80 µm, respectively. The working distance and the confocal parameter of the OCT is 1.3 mm and 0.65 mm, respectively, which was optimized for imaging coronary arteries^1^. Since the FLIm excitation beam was guided by the multi-mode cladding, it was less affected by confocal effect than the OCT. Thus, the spot size of the FLIm excitation beam remained relatively constant within the confocal parameter of the OCT beam.

**
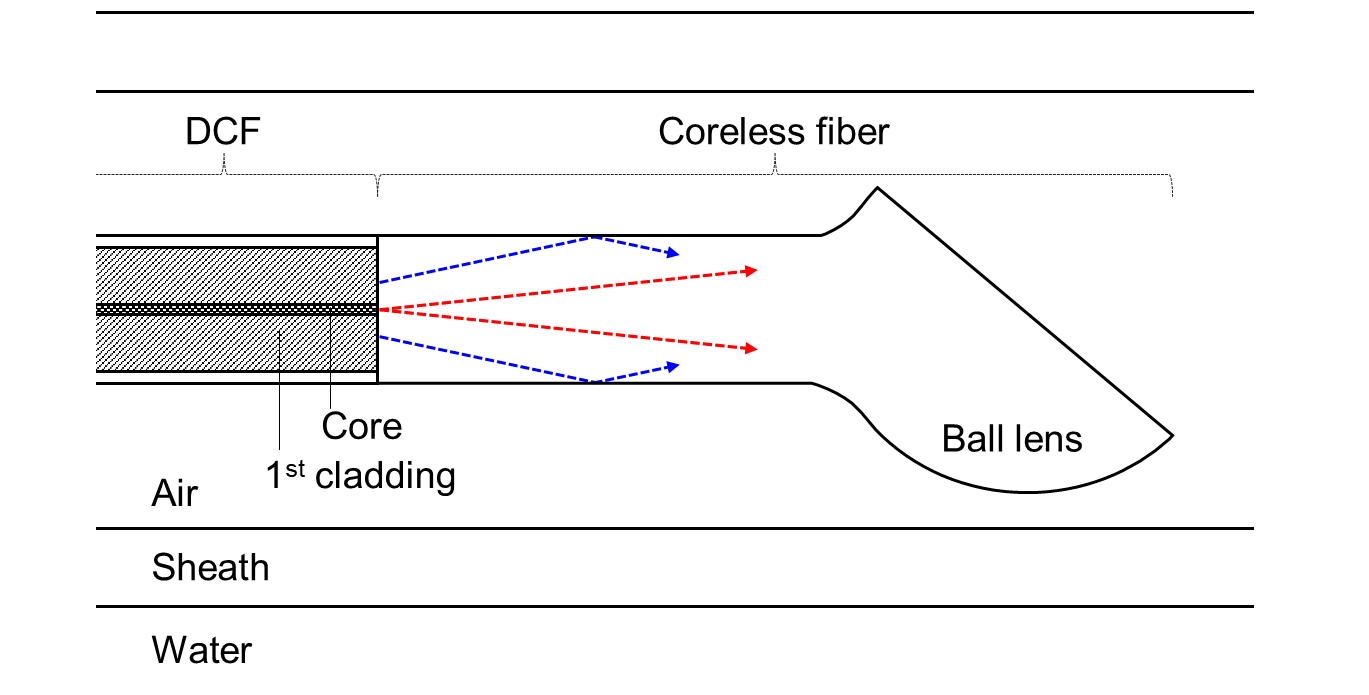
**

Supplementary Figure S2 Schematic diagram of the ball lens imaging core. Red and blue dotted lines represent the OCT and FLIm beams through the core and first cladding of the DCF, respectively.

**Validating accuracy and precision of FLIm using standard fluorophores**

In order to validate the accuracy and precision to measure fluorescence lifetime, multispectral fluorescence lifetimes were measured using standard fluorophores. Coumarin 120 and Rhodamine 6G dissolved in ethanol were used as standard fluorophore samples. The fluorescence lifetimes were measured by helically scanning with the catheter immersed in the two solution samples. 100 pixels were randomly sampled from the measured fluorescence lifetime, and mean and standard deviation were calculated using the sampled data. Supplementary table 1 shows the measured fluorescence lifetimes in each channels (mean ± standard deviation). The difference of the measured fluorescence lifetime was less than 1.38% compared to the literature^2, 3^. The precision, defined as the standard deviation, was less than 0.05 ns without any averaging.

Supplementary Table 1 Comparison of the measured fluorescence lifetime of standard fluorophore with literature

| Standard fluorophore | Fluorescence lifetime in ^2, 3^ (ns) | Measured fluorescence lifetime (ns) | | |
| --- | --- | --- | --- | --- |
|  |  | Channel 1  (390/40 nm) | Channel 2  (452/45 nm) | Channel 3  (542/50 nm) |
| Coumarin 120 in ethanol | 3.64 | 3.61±0.05 | 3.59±0.05 | - |
| Rhodamine 6G in ethanol | 3.99 | - | - | 3.97±0.05 |

**Removing autofluorescence artifacts caused by the doped silica of the DCF**

When collecting fluorescence emission pulse sequences from a sample using the IV-OCT/FLIm imaging catheter, unwanted autofluorescence emitted by the germanium-doped core and low-index cladding of the DCF is also detected (Supplementary Fig.S3(a)). The fluorescence lifetime calculations can be significantly distorted unless the artifacts are properly removed. Fortunately, the autofluorescence artifacts mainly originate from the proximal end of the DCF, whereas the sample is located near the distal end of the DCF, which has a length of about 1.6 m. Therefore, the autofluorescence artifacts and the sample signal do not overlap with each other, and the artifacts can easily be excluded from the signal. Because the length of the DCF is fixed, the artifacts always arise at the same relative positions in time (red solid lines in Supplementary Fig.S3(a)). In addition, the pulse widths of the artifacts are always the same because the source of the artifacts is also fixed. Under those assumptions, we first found the position of the maximum of each pre-determined region of interest with the artifacts (red solid lines in Supplementary Fig.S3(a)) and then set the fixed number of values around the maximum position to zero for all fluorescence lifetime measurements. By removing the artifacts, the fluorescence lifetime could be successfully calculated despite the presence of the artifacts (Supplementary Fig.S3(b)).


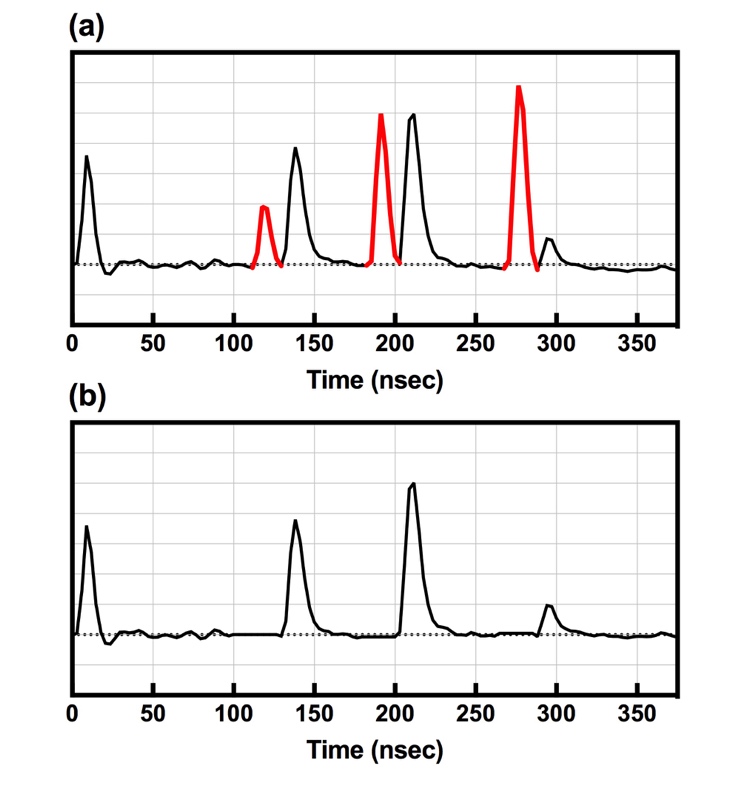


Supplementary Figure S3. (a) Representative fluorescence emission pulse sequence waveform (black solid lines) with autofluorescence artifacts from the doped material (red solid lines) in the DCF. The artifacts can severely degrade the measurement performance and should thus be removed before calculating the fluorescence lifetime. (b) Representative fluorescence emission pulse sequence waveform after removing the autofluorescence artifacts.

**Fluorescence lifetime characteristics independent of fluorescence intensity**

In catheter-based fluorescence imaging, the fluorescence intensity decreases as the distance between the catheter tip and the fluorophore increases. This intensity variation might cause misinterpretations in image analysis, especially when intensity itself is an important factor. However, fluorescence lifetime, which is an inherent characteristic of fluorophores, is independent of intensity. We have confirmed that characteristic of fluorescence lifetime using our IV-OCT/FLIm catheter system. We acquired the fluorescence of a lens tissue that emits autofluorescence when irradiated with UV light. The catheter was obliquely placed on the lens tissue, and the lens tissue was helically scanned to measure the fluorescence intensity and lifetime at various distances. We randomly sampled 100 pixels from the fluorescence intensity and lifetime measured in channel 2. The fluorescence lifetime is plotted as a function of fluorescence intensity in Supplementary Fig.S4. The fluorescence lifetime was constant regardless of fluorescence intensity, even though the fluorescence intensity varied depending on the distance.


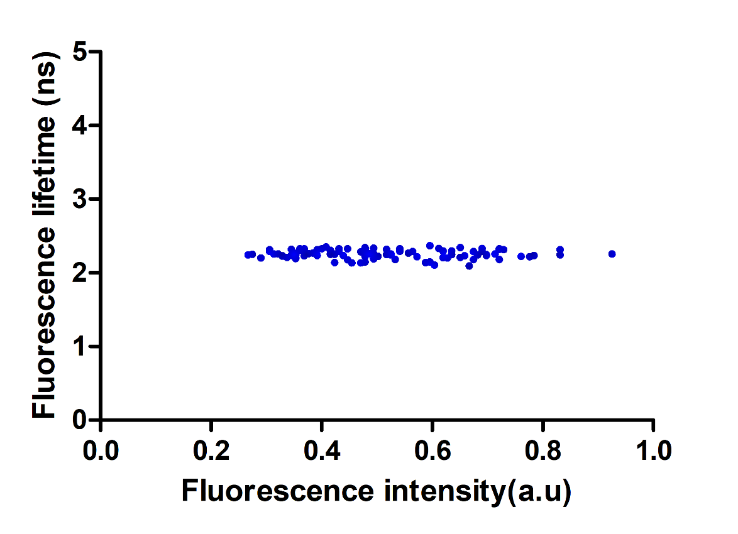


Supplementary Figure S4. Plot of fluorescence lifetime as a function of fluorescence intensity. The fluorescence lifetime obtained with the IV-OCT/FLIm catheter was constant regardless of the fluorescence intensity.

**References**

1. Cho HS*, et al.* High frame-rate intravascular optical frequency-domain imaging in vivo. *Biomed Opt Express* **5**, 223-232 (2013).

2. Pal H, Nad S, Kumbhakar M. Photophysical properties of coumarin-120: Unusual behavior in nonpolar solvents. *The Journal of chemical physics* **119**, 443-452 (2003).

3. Magde D, Rojas GE, Seybold PG. Solvent dependence of the fluorescence lifetimes of xanthene dyes. *Photochemistry and Photobiology* **70**, 737-744 (1999).
